# Impact of COVID-19 emergency on the psychological well-being of susceptible individuals

**DOI:** 10.1038/s41598-022-15357-6

**Published:** 2022-07-01

**Authors:** Angela Stufano, Guglielmo Lucchese, Benjamin Stahl, Ignazio Grattagliano, Liliana Dassisti, Piero Lovreglio, Agnes Flöel, Ivo Iavicoli

**Affiliations:** 1 Interdisciplinary Department of Medicine, Section of Occupational Medicine ‘EC Vigliani’, University of Bari, Policlinico, Piazza G. Cesare 11, 70124 Bari, Italy; 2 Department of Neurology, Universitätsmedizin Greifswald, Greifswald, Germany; 3 Department of Neurology, Charité Universitätsmedizin Berlin, Berlin, Germany; 4 Max Planck Institute for Human Cognitive and Brain Sciences, Leipzig, Germany; 5 Faculty of Science, Medical School Berlin, Berlin, Germany; 6 Department of Education Science, Psychology, Communication Science, University of Bari, Bari, Italy; 7 Department of Public Health, Section of Occupational Medicine, University of Naples Federico II, Naples, Italy

**Keywords:** Health occupations, Psychology

## Abstract

The current pandemic has exerted an unprecedented psychological impact on the world population, and its effects on mental health are a growing concern. The present study aims to evaluate psychological well-being (PWB) during the COVID-19 crisis in university workers with one or more diseases likely to increase the risk of severe outcomes in the event of SARS-CoV-2 infection, defined as susceptible. 210 susceptible employees of an Italian University (aged 25–71 years) were recruited during the COVID-19 second wave (October–December 2020). A group comprising 90 healthy university employees (aged 26–69 years) was also recruited. The self-report Psychological General Well Being Index (PGWBI) was used to assess global PWB and the influence on six sub-domains: anxiety, depressed mood, positive well-being, self-control, general health, and vitality. We applied non-linear dimension-reduction techniques and regression methods to 45 variables in order to assess the main demographic, occupational, and general-health-related factors predicting PWB during the COVID-19 crisis. PGWBI score was higher in susceptible than in healthy workers, both as total score (mean 77.8 vs 71.3) and across almost all subscales. Age and jobs involving high social interaction before the pandemic were inversely associated with the PWB total score, general health, and self-control subscores. The current data suggest no decline in PWB during the second wave of COVID-19 health emergency in susceptible individuals of working age. Critically, higher risk for mental-health issues appears to be inversely related to age, particularly among individuals deprived of their previous level of social interaction at work.

## Introduction

Well-being can be generically defined by each person's degree of positive feelings experienced and overall perceptions of life^1^. More than merely as the absence of negative circumstances such as illness or emotional distress, general well-being should be considered as the sum of positive mental and physical health aspects, thus demanding more holistic approaches to disease prevention and health promotion^2^. Psychological well-being (PWB) is usually conceptualized as the combination of positive affective states and functioning with optimal effectiveness in life, referred to as the hedonic and eudemonic perspectives, respectively^3^. Health emergencies, such as epidemics, can have detrimental and long-lasting psychological consequences on the population’s well-being rates, as seen during the SARS-CoV-1 outbreak^4,5^.

Along with the medical challenges and the uncertainty regarding the management of the healthcare system, the COVID-19 pandemic has had a substantial psychological impact on the world population, and its effects on general mental health are raising increasing concerns. The policies adopted by governance, such as social distancing and lockdown measures, can cause social isolation and loneliness, variables known to decrease well-being and increase the risk of depression^6^. While these steps may be critical to mitigate the spread of this disease, they undoubtedly have consequences on mental health and well-being in both the short and long term^7^.

A variety of factors were associated with higher risks of psychiatric symptoms and low PWB of the general population during the COVID-19 pandemic, such as female gender, higher education, current or past chronic illnesses, and higher social media exposure^8^. Accordingly, several studies were focused on the development of stress reactions within risk groups, or on the common psychological effects fueled by the pandemic^9^. Italy was the first European country that had to face the pandemic, and here, high rates of detrimental effects on mental health were found in the general population already after three to four weeks into lockdown measures, independently of previous mental illness or childhood trauma^10^.

The COVID-19 pandemic could induce psychological stress in susceptible populations beyond the stress also suffered due to their disease, as they are disproportionately at risk of severe symptoms in the event of SARS-CoV-2 infection^11^. In fact, those with specific underlying health conditions are more susceptible to serious health effects of COVID-19, and should therefore implement social and workplace distancing to a greater degree to avoid being infected; this may cause a more severe impact on their well-being of the pandemic^12^. In Italy, in particular, the definition of "susceptible worker" has been introduced, indicating employees with an increased risk of developing severe forms of COVID-19 due to age and disabilities, or condition resulting from immune-depression, cancer or immune impairment due to life-saving therapies. The pandemic could also have serious mental health effects in workforce members obliged to stop working due to lockdown or other preventive policies.

In short, previous research strongly suggests the need to make an accurate and timely assessment of the magnitude of the PWB outcome in populations exposed to the COVID-19 pandemic, as compared to the previous health status, although to date, no study has specifically investigated susceptible individuals of working age.

The aim of the current study was to assess rates of personal PWB in a group of susceptible workers in an Italian University during the COVID-19 emergency, with the aim of gaining evidence that could potentially inform subsequent research strategies and overall mental health delivery.

## Methods

### Participants and study design

A cross-sectional survey was conducted on the employees of an Italian University (Bari, Apulia) that met the criteria to have applied during the period 01 October–22 December 2020 for inclusion in the exceptional occupational health surveillance for susceptible workers. In fact, Italian Law Decree No. 34 of May 19, 2020 had introduced specific health surveillance for workers deemed susceptible to serious health damage from COVID-19 infection due to underlying health conditions. The occupational physician was by law assigned the task of identifying any additional appropriate preventive measures to allow them to continue working safely, including those working online from home. Exclusion criteria for enrollment in the study comprised: a previous documented SARS-CoV-2 infection, pre-existing psychiatric disorders necessitating pharmacological treatment, working abroad during the study period, and lack of susceptibility according to the criteria described below.

The workers recruited in the present study held the position of professor (full professor, associate professor, senior researcher and tenure-tracked researcher), technical clerk, and administrative employee. The professors deal with teaching and scientific research, the technical clerks carry out technical support tasks for scientific research, such as laboratory and informatics activities, while the administrative employees perform front office activities for professors and students, support the teaching activities, collect and compile documents and records relating to professors and students at the University. All University employees had to move to remote work as from the beginning of the COVID-19 health emergency on March 11, 2020.

A group of healthy University employees was also recruited within the activities of the Occupational Medicine Section. The subjects included in this group were chosen according to sex and age, to obtain a distribution of these two variables similar to that observed in the study population.

During the study period, the following restrictive measures of public health were implemented: at least one meter distancing between people in all indoor and outdoor places; use of masks mandatory in all indoor places and in all outdoor places where distancing was not possible; sports events, individual and team sports competitions, activities in gyms, swimming pools, wellness centres and spas suspended, both in public and private places; performances open to the public, including open-air performances, in theatres, concert halls and cinemas, discos and dance halls suspended; celebrations of any kind, including those associated with civil or religious ceremonies such as weddings prohibited; restaurant and other catering activities only allowed from 5 am to 6 pm.

The study protocol was approved by the Ethics Committee of Bari University Hospital (Protocol n. 0007236). Participants were fully informed about the aims and procedures of the study and informed consent was obtained before starting the survey. Participation was entirely voluntary, and participants could withdraw from the study at any time. The study was conducted according to the criteria set by the declaration of Helsinki.

### Data collection and assessment of susceptibility

A questionnaire was designed to collect general data about employees, including gender, age, body mass index (BMI), occupational tasks performed prior to the transition to online working (e.g. teaching, research, administrative), smoking and alcohol consumption, and to assess participants' preexisting pathological conditions for the definition of susceptibility, because considered to be associated with an increased risk of serious outcomes in case of SARS-CoV-2 infection. In this regard, all pathological conditions identified by Clark et al.^13^, using prevalence figures from the Global Burden of Diseases, Injuries, and Risk Factors Study 2017 to estimate the global numbers of individuals at increased risk of severe COVID-19, were taken into account. Particularly, the questionnaire asked for the presence of respiratory diseases, vascular disease, neoplastic, thromboembolic and ischemic neurological diseases in the previous 5 years, inflammatory, degenerative and congenital neurological diseases, diabetes, chronic kidney and liver diseases, autoimmune diseases, tuberculosis and HIV infections, condition of immunosuppression both congenital and acquired (see “Questionnaire” in Supplementary Material).

Susceptibility to COVID-19 was assessed for each employee by an adapted version of the COVID age tool. This risk model was modified according to the recent scientific data used to make estimates of personal susceptibility to COVID-19 according to gender, age and diseases described above, taking into account the national recommendations^12,14^. However, the presence of one or more of the diseases described above, that would increase the risk of severe outcomes in the event of SARS-CoV-2 infection, defined the worker as susceptible.

The occupational risk assessment for SARS-CoV-2 infection was carried out for each work activity performed by University employees before the COVID-19 health emergency and the transition to online working. The methodological approach implemented to estimate the risk, was that adopted by the national Scientific Committee and set up by the Italian government to provide action-oriented policy advice on the COVID-19 emergency^15^. Occupational risk assessment was made on the basis of the three parameters: exposure probability, proximity index and aggregation factor. The first parameter measures the probability, due to the work activity, of coming in contact with infected people, while the proximity index measures physical proximity to others during work activities, adapting the proximity perception indicators defined by O*Net8 methodology to the Italian context (O*NET 24.2 database https://www.onetcenter.org/dictionary/24.2/excel/). The aggregation factor quantifies the degree of social aggregation connected to the job, ranging from the scant presence of third parties to large aggregations not easily controlled by specific procedures. The probability of exposure is assumed to be uniform across the University work context, while the proximity and aggregation indexes describe five different conditions associated with increasing values of the indexes.

### Assessment of individual psychological well-being

Individual PWB was assessed by the Italian version of the Psychological General Well-Being Index (PGWBI), a 22-items self-reported questionnaire aimed at measuring subjective well-being or discomfort within the last 28 days^16,17^. The items reflect the six subscales: anxiety, depression, positive well-being, self-control, general health, and vitality, including 5, 3, 4, 3, 3, and 4 items, respectively. A 6-point Likert scale (from 0 to 5) provides subscale and total scoring that can reach a maximum of 110 points, where higher scores indicate better PGWB^18^. PGWBI scores have been grouped into the following divided categories: 0–60 Severe Distress, 61–71 Moderate Distress, 72–92 No Distress, and 93–110 PWB^19^. The Italian version of the questionnaire, validated in 2002 in a non-clinical population, has acceptable reliability and internal consistency, with a Cronbach’s alpha coefficient for each scale ranging from 0.61 to 0.85, and item-scales correlations ranging from 0.43 to 0.67^17^.

### Statistical analysis

The analyses were performed with SPSS 25 (IBM Corp., Armonk, NY, USA). Continuous variables were expressed as mean and standard deviation (SD), and categorical variables as raw frequency and percentage.

The relationship between general and job characteristics, the presence of one or more diseases and the PGWBI scores was investigated by applying dimensionality reduction methods, followed by linear regression. Non-linear principal component analysis (NLPCA), also known as categorical principal component analysis (CATPCA), was used to map the variables onto a lower dimensional space with the advantage of eliminating any redundancy and minimizing multicollinearity. The CATPCA allows linear PCA to be extended to ordinal and nominal categorical variables while exploring possible non-linear relationships and is widely employed in biomedical and social sciences^20,21^. Age, BMI, weekly alcohol units, age at first use of tobacco, years of smoking, daily number of cigarettes, pack years (PY), systolic and diastolic body pressure, total cholesterol, and high-density lipoprotein levels (HDL) were considered numeric variables and discretized by multiplication, all other variables were quantified as nominal. Solutions were computed for a four to eight-dimension range, applying non-parametric bootstrap to assess significance of the loadings on the components. The six-dimension solution was adopted for analysis because all the confidence intervals of the loadings on the seventh component included the value zero, and the first six components explained ≈47% of the total variance, indicating adequate fit^20^. Dimensions (also defined as components) were then VARIMAX rotated^20^.

Finally, we performed linear regression, applying each of the six series of CATPCA scores, one for each rotated component, as predictors for each of the six PGWBI subscales and of the total scores, for a total of seven regression tests for each component, then corrected with the Bonferroni method^21^. A p-value lower than 0.05 was considered statistically significant for all the statistical analyses.

## Results

General characteristics, and number of diseases of the study participants are reported in Table 1. At the time of the study, the whole University workforce numbered 2830 employees, including 1521 academic (271 full professors, 524 associate professors, 425 senior researchers and 301 tenure-tracked researchers) and 1309 non-academic workers (627 technical clerks and 682 administrative employees). Of the overall study population of 248 employees, 210 were included in the final cross-sectional analysis because voluntarily completed the survey, amounting to a global response rate of 84.7%.

**Table 1:** General characteristics, job and number of diseases in the study subjects.

Characteristics	N. (%)	Mean ± SD	Range
Age (years)	210	59.2 ± 9.9	25–71
BMI (Kg/m^2^)	210	26.2 ± 6.0	16.4–43.8
Obesity (BMI ≥ 30)	35 (16.6%)		
**Gender**			
Male	103 (49.0%)		
Female	107 (51.0%)		
**Job (study subjects/whole university employees)**			
**Professor**	101 (48.1%) / 1521 (53.7%)		
Full professor	39 (18.6%) / 271 (9.6%)		
Associate professor	53 (25.2%) / 524 (18.5%)		
Tenure-tracked researcher	5 (2.4%) / 301 (10.6%)		
Senior researcher	4 (1.9%) / 425 (15.0%)		
**Technical clerk**	24 (11.4%) / 667 (23.6%)		
**Administrative employee**	85 (40.5%) / 682 (24.1%)		
**Smoking habit**			
Non smokers	130 (61.9%)		
Ex smokers	38 (18.1%)		
Current smokers	42 (20.0%)		
Alcohol consumption (weekly units)	210	2.6 ± 3.9	0–14
**N. diseases**			
1	107 (50.9%)		
2	48 (22.9%)		
3	31 (14.8%)		
4	18 (8.6%)		
5	3 (1.4%)		
6	3 (1.4%)		

The University employees who completed the survey had a mean age of 59.2 ± 9.9 years and the number of male and female subjects was similar (49% vs 51%); the BMI was 26.1 ± 6.0 (mean ± SD), while in 16.6% of the participants, values were ≥ 30, falling in the obesity range^22^. As regards the job, the study population was divided into professors (48.1%), technical clerks (11.4%) and administrative employees (40.5%). In terms of smoking habit, 20.0% of the subjects were current smokers, 18.1% reported a past smoking habit and 61.9% had never smoked. The study population reported a mean weekly alcohol unit consumption of 2.6 (range 0–14 units). The number of diseases referred by the study subjects during the exceptional health surveillance visit ranged between 1 (50.9%) and 6 (1.4%).

Of 203 healthy university employees considered, 90 were included in the final cross-sectional analysis because not referring any susceptibility to severe forms of COVID-19 and voluntarily completed the survey. This group showed a similar age (mean 57.1 ± 10.0 years) and sex (female 51%) distribution than the study population.

The total and subscale scores for the overall employees are reported in Table 2. The total mean score (77.8) was located in the area of “No distress” and showed higher value than that reported for the healthy workers (mean 71.3), as for nearly all the subscales scores (p < 0.01 or p < 0.001). On the contrary, similar values compared to the mean reference scores for the Italian population (total 78.0) were observed, with only the general health mean subscore (10.1) significantly lower (p < 0.001) than the mean reference levels (11.1)^17^.

**Table 2:** PGWBI scores for each subscale in the study population, in the healthy workers, in the Italian population validation sample (Grossi et al., 2002) and in Rossi et al. (2021) population sample.

Subscales	Research sample (N. 210)		Healthy workers (N. 90)		PGWBI validation sample (N. 1129)		Rossi et al. sample (N. 2013)
	Mean ± SD	Range	Mean ± SD	Range	Mean ± SD	Range	Mean ± SD
Anxiety	17.8 ± 4.2^b^	4–25	15.2 ± 5.0^b^	1–24	17.3 ± 4.9	1–25	16.1 ± 4.7
Depressed mood	12.5 ± 2.2^a^	2–15	11.7 ± 2.9^a^	2–15	12.4 ± 2.6	0–15	8.5 ± 1.5
Positive well-being	12.1 ± 3.7^a^	2–19	11.0 ± 3.8^a^	0–19	11.8 ± 4.0	0–20	9.9 ± 3.2
Self control	11.9 ± 2.7^a^	2–15	11.1 ± 2.8^a^	1–15	11.8 ± 2.6	1–15	12.2 ± 3.2
General health	10.1 ± 3.1^c^	3–15	10.4 ± 2.7	3–15	11.1 ± 3.0^c^	0–15	7.4 ± 1.8
Vitality	13.5 ± 3.7^b^	3–20	11.9 ± 4.0^b^	0–20	13.4 ± 4.0	0–20	10.4 ± 2.7
Total	77.8 ± 17.4^a^	17–106	71.3 ± 19.9^a^	26–104	78 ± 17.8	8–110	64.5 ± 13.1

Research sample vs healthy workers: ^a^p < 0.01; ^b^p < 0.001.

Research sample vs PGWBI validation sample: ^c^p < 0.001.

A six-dimension solution for the CATPCA was adopted and the variance accounted for all the components and dimensions. Variables loading on the component with coefficient absolute values equal to or higher than ± 0.4 were considered^20^. Age, the job and a cluster of variables related to the working activities performed before the COVID-19 pandemic (administrative tasks, front office activity with student and colleagues, proximity index, research and teaching tasks) loaded with a higher coefficient than ± 0.4 (Table 3). Therefore, contributing most heavily to the first component, and explaining the largest amount of variance in the data (13.2%), these variables were considered the ones affecting PWBI the most in our analysis (Table 3).

**Table 3:** Variables loading with absolute values ≥  ± 0.4 on the first principal component (variance accounted for 13%), the only one showing significance as a predictor of PWBI in the regression analysis.

Variable	Loading*
Job	0.970
Administrative tasks	0.939
Front office tasks	0.743
Proximity Index	0.521
Age	− 0.715
Research tasks	− 0.759
Teaching tasks	− 0.956

*Index of the correlation of the original variables with the first principal component.

The second component, consisting of a combination of smoking habit, age at first use of tobacco products, years of smoking, number of daily cigarettes, and pack-years, accounted for 9.4% of the total variance. The third component, explaining 8.3% of the data variance, included the number of cardiac and of total diseases. The fourth component, accounting for 6.0% of the variance in the data, was correlated with the number of respiratory diseases, occurrence of dyspnea and BMI. The fifth component, namely a combination of alcohol drinking habit, the alcoholic beverage of choice, and the number of weekly alcohol units, contributed 5.2% of the total variance. Neoplastic diseases and treatment made up the sixth component, which accounted for 4.5% of the variance. None of these variables was loaded with a coefficient higher than ± 0.4.

In the regression analysis using the component scores as predictors of the PGWBI, a significant regression equation was found only with the first component for the total score (F(6,203) = 3.77, p = 0.001, R^2^ = 0.10), as well as for the general health (F(6,203) = 7.75, p < 0.001, R^2^ = 0.19) and self-control (F(6,203) = 3.18, p = 0.005, R^2^ = 0.86) subscores (Table 4).

**Table 4:** Regression analysis of the first component on the different subscales and the total score in the study population.

	First component		
Subscales	F(6,203)	p	R^2^
Anxiety	–	NS	–
Depressed mood	–	NS	–
Positive well-being	–	NS	–
Self control	3.18	0.005	0.86
General health	7.75	< 0.001	0.19
Vitality	–	NS	–
Total	3.77	0.001	0.10

*NS* not signifcant.

**Figure 1:**
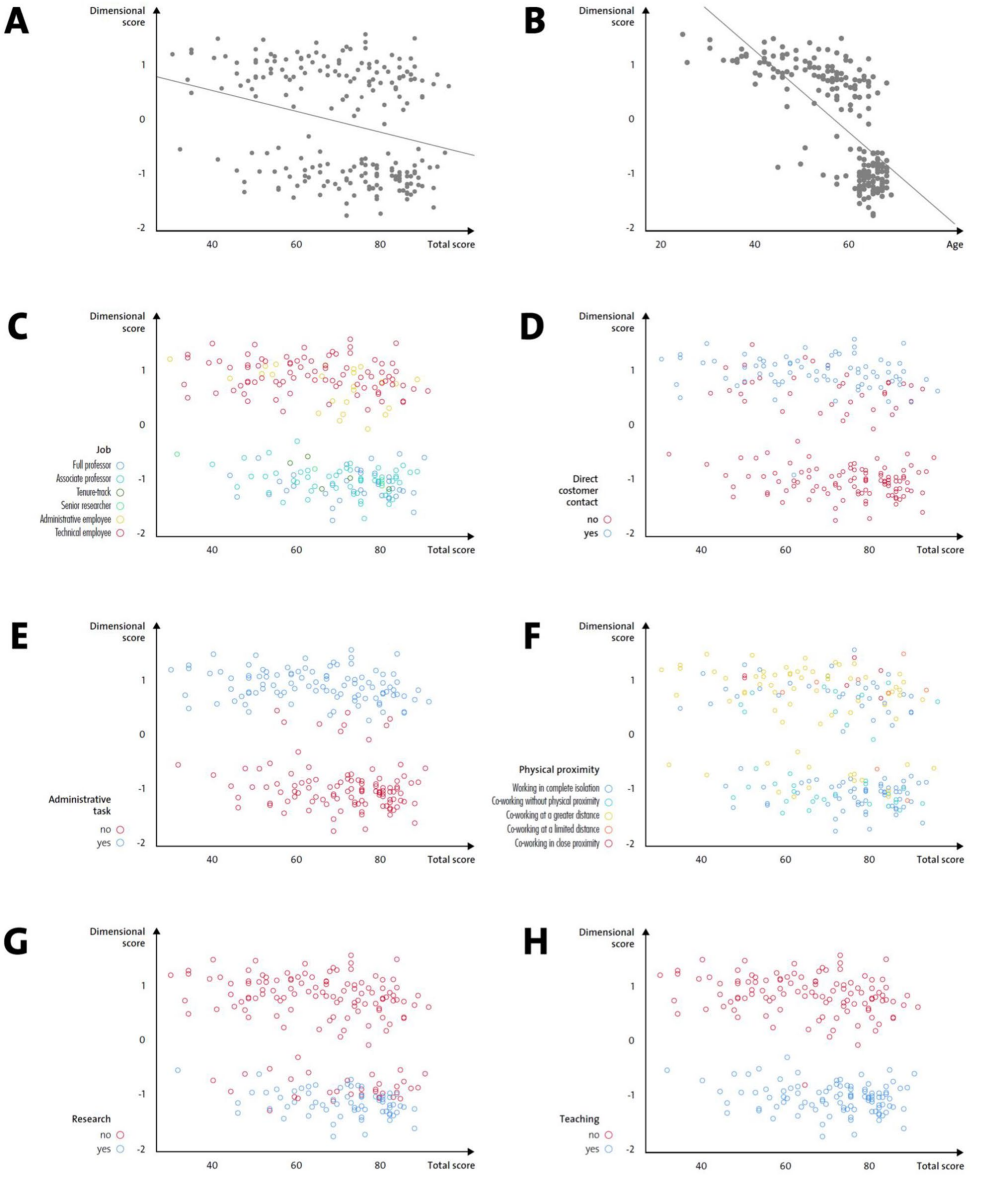
(**A**) Regression analysis between the first component (dimensional score) and the dependent variable total well-being scores. (**B**) Regression analysis between the first component (dimensional score) and age, the only linear independent variable contributing to the first component. (**C**–**H**) Relationship between the independent variables (job, direct contact, administrative task, physical proximity, research and teaching activity) identified by the PTCA as contributing to the first dimension and the dependent variable total well-being score.

**Figure 2:**
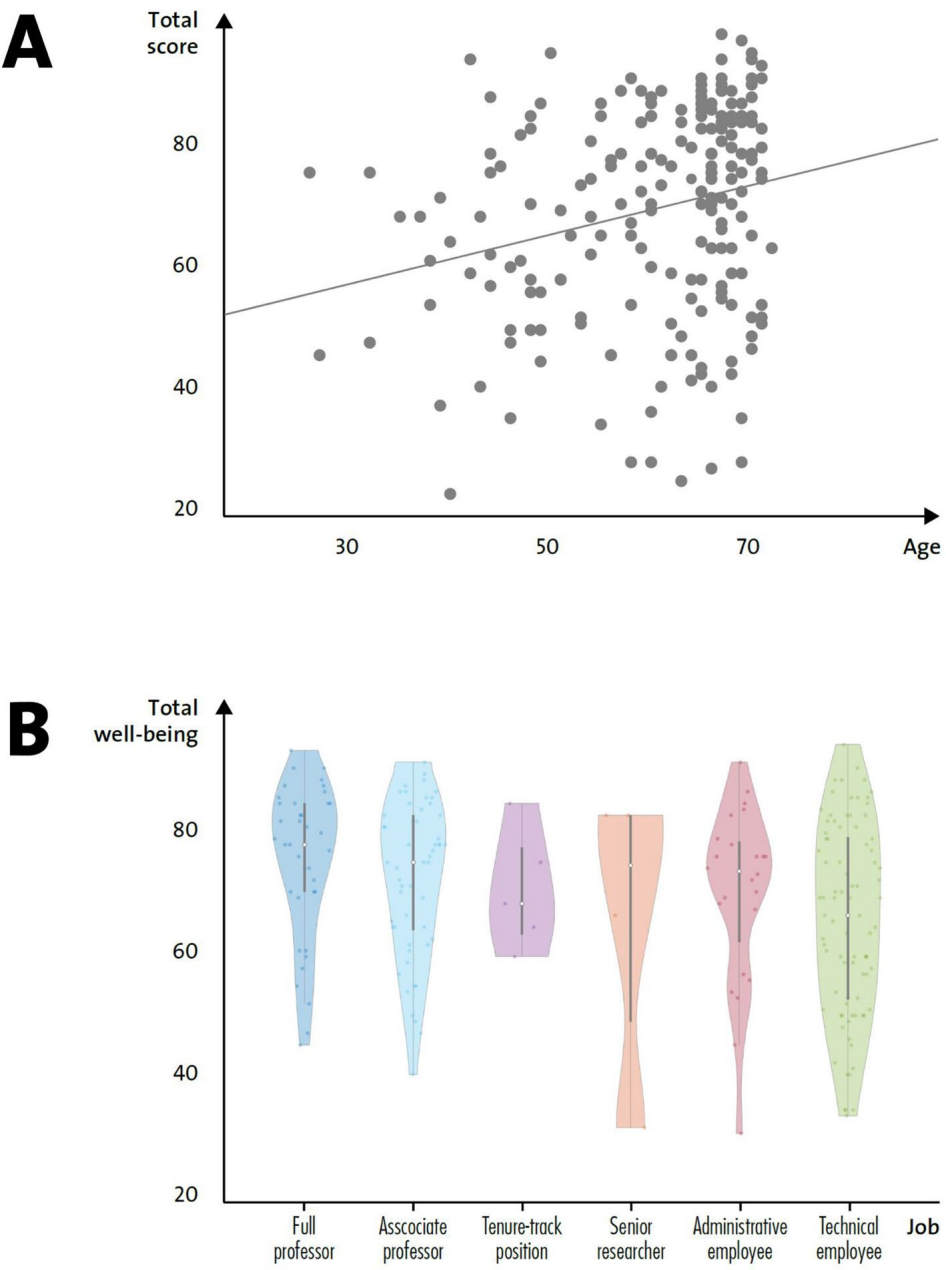
(**A**) Relationship between the independent variable age and the dependent variable total well-being score. (**B**) Total well-being score expressed as function of job; the violin plots show the median, the values comprised between the 2nd and 3rd quartile and the total range, as well as kernel density estimates.

The first rotated component negatively predicted total well-being (Fig. 1, Panel A) as well as general health and self-control. Among the variables mainly contributing to the first component, age was the only continuous variable, and showed a negative loading (Table 3). This finding indicated an inverse association with the component, as shown in Fig. 1, panel B, suggesting that total well-being (Fig. 2, Panel A), general health, and self-control were greater in older individuals.

The remaining variables listed in Table 3 were considered as nominal in the CATPCA and, therefore, their relationship with the first rotated component cannot be assumed to be linear and is more easily interpreted graphically, as shown in Fig. 1 (Panel C–H)^20,23^. The first rotated dimension resulting from the CATPCA accurately distinguished between academic (professor) and non-academic (technical clerk or administrative employee) jobs; the well-being scores of employees with academic roles were preferentially clustered at the higher range of the scale (Fig. 1, Panel C and Fig. 2, Panel B). A similar trend was observed for the other job-related variables. Well-being scores of employees with no direct contact with students or colleagues, no administrative tasks, a low proximity index and research and/or teaching tasks, were clustered at the higher range of the scale (Fig. 1, Panels D–H).

## Discussion

Our findings show that the COVID-19 pandemic does not seem to have negatively affected the general PWB of susceptible subjects of working age. The well-being rates observed in the study population, moreover, do not appear to be related to the specific diseases from which these workers suffer, or to older age. On the contrary, increasing age seems to be a factor related to higher total well-being, general health, and self-control scores. Moreover, the well-being scores of employees working before the COVID-19 pandemic in higher proximity and direct contact with colleagues and students were clustered at the lower range of the scores.

In our study, PWB showed higher values in comparison to the healthy university workers and similar to the Italian normative values, however obtained on a younger population^17^. PWB in susceptible workers is in line with a survey conducted during COVID-19 pandemic on the healthy Italian population, which through the use of the International Physical Activity Questionnaire (IPAQ) and the PGWBI short version showed the lack of alteration in the PWB levels of the subjects interviewed^24^. However, our findings in susceptible workers seem to be in contrast with the available literature related to the Italian COVID-19 emergency which describes the significant negative impact of lockdown and containment measures on psychological health, and a reduced general PWB in the Italian population^25^. Particularly, the scores observed in our study, remarkably higher than those reported by Rossi et al. (total score 77.8 vs 64.5), could be related to the context in which our survey was performed, i.e. the beginning of second wave of COVID-19 emergency. After a decrease of detected cases in summer, Europe was faced with the appearance of a COVID-19 wave and, unexpectedly, the number of infected cases resulted to be lower in Italy than in other European countries, probably because of policies that helped to limit the virus spread^26^. This might have had a beneficial effect on psychological health on our study sample. In addition, the survey was conducted in southern Italy, where the impact of the COVID-19 health emergency was strongly contained compared to that experienced in the north of the country, while the study sample described by Rossi et al.^25^ was mainly related to a Northern population (69%)^27^.

The COVID-19 pandemic has led to the identification of a new form of vulnerability in society and work settings. In the context of the COVID-19 emergency, in fact, a frailty condition was attributed to a new population of susceptible individuals, suffering from conditions posing an increased risk of developing severe or fatal forms of COVID-19, although these conditions do not necessarily cause a significant reduction in the individual ability to carry out daily and work activities. No role of specific diseases emerged from our analysis. In fact, although a previous survey showed that physical symptoms and poor physical health were factors associated with the severity of psychiatric symptoms in the workforce after protracted lockdown, the impact of a particular disease on PWB does not seem to be associated with its clinical severity and cannot be directly inferred by a clinical evaluation^28,29^. Moreover, previous research into past outbreak situations identified the fear of infection as a common risk factor, with an impact on individual mental health and well-being, particularly in individuals with pre-existing diseases. A recent survey in the US population also demonstrated that more susceptible individuals, particularly those with preexisting health conditions, suffered a disproportionately greater impact of COVID-19 restrictions on their mental health^30^. However, the lack of correlation between the number of diseases and the levels of PWB observed in our study seems to suggest that the PWB scores are presumably influenced by other factors more than by the pathological conditions of the recruited subjects. In fact, these workers had diseases that, before the COVID-19 pandemic, did not prevent them from working, whereas they acquired a significant weight as conditions associated with the development of severe outcomes in the event of SARS-CoV-2 infection. Therefore, it can be assumed that it is not the pathological conditions per se that affect the well-being levels of these subjects.

The positive association between well-being and age seems to be related to the subjective well-being paradox, defined as the absence of a strong decline in general well-being in the face of possible age-related health decline^31,32^. The greater well-being in older subjects seems to be mainly a result of adaptation, emotional regulation, and accommodation strategies, such as rescaling goals and adjusting aspirations to the given situation. Our results show that the "age paradox" may play a relevant role during the COVID-19 health emergency, and is preserved even in the presence of clinical conditions associated with an increased susceptibility in the event of SARS-CoV-2 infection. This finding is in line with previous research focused on the quality of life in susceptible subjects before and during the pandemic, that showed that greater age was directly associated with better outcomes^25,33^.

In addition to age, some variables related to the individual occupational tasks performed before moving to online home working at the beginning of the pandemic were associated to the total well-being score and general health and self-control subscores. Particularly, employees who performed tasks associated with higher proximity and work in direct contact with students and colleagues were clustered at the lower range of the scale, indicating that susceptible employees deprived of social interactions in the workplace might suffer more than those performing job activities that do not include high interaction with colleagues and students. Social distancing may worsen mental health problems, especially within highly collectivist cultures like the Italian one, in which social connections are valued very deeply^34^. A recent study pointed out that workers employed in businesses relying heavily on face-to-face communication or close physical proximity are particularly vulnerable to social distancing interventions. An effective interpersonal work relationship is a critical factor for job satisfaction, and the loss of these interactions may therefore be associated with lower levels of general PWB. This could be due to a brain-to-brain group synchrony and to a “joint attention”, which requires eye contact and the exchange of glances (mutual gaze), that cannot be matched when working in videoconferencing^35^. Socializing and social participation, especially at work, were found to crucially contribute to maintaining well-being after disasters^36^. In fact, the socio-emotional selectivity theory, which proposes the use of social networks as a buffer against negative experiences, could also contribute to explain why well-being decreases in individuals deprived of their social interaction^37^.

Our study has some limitations. Firstly, the sample is representative of a specific socio-economic, geographic, and occupational context and the findings warrant replication in different cultural settings. Secondly, variables related to the actual working activity performed in remote mode, which could have affected the PWB of the subjects, were not evaluated. The study, however, aimed to assess the impact of the COVID-19 pandemic on general, not specifically occupational well-being, that cannot be investigated by the use of the PGWBI. Finally, the diseases driving the susceptible condition, although assessed using the same criteria, were highly heterogeneous, and this could be the reason why the analysis did not identify a differential impact of specific diseases on PWB. Despite these potential limitations, our study has several strengths. To the best of our knowledge, this is the first study to investigate the effect of the COVID-19 pandemic on the PWB of susceptible individuals of working age, whose need to adopt strict prevention measures could have caused extensive changes in daily routine and working life. This study, moreover, investigated susceptible workers that had a normal life before COVID-19, giving a further contribution to understand the PWB during the pandemic. Moreover, it is one of the few studies on the psychological impact of COVID-19 relying not on the use of online tools, but on personal assessment by trained medical personnel. Thus, we were able to collect information on the clinical status of the participants, excluding those who referred current and past psychiatric disorders or treatment with psychotropic drugs, and allowing the participants to ask questions when in doubt about the meaning of an item, in accordance with the guidelines for the administration of PGWBI^17^. It is important that the PGWBI questionnaire is a validated measure of health-related quality of life, widely used in clinical trials and epidemiological research to provide an overall assessment of health and self-perceived PWB in individuals with chronic conditions^38–40^. Finally, the use of CAPTCA analysis allowed an unbiased evaluation of a large number of factors, while maintaining methodological rigor and making appropriate corrections for multiple comparisons.

## Conclusion and outlook

The results presented here have considerable relevance on much-needed large-scale preventive measures for mental health conditions in the general population in order to support social connections and individual resilience. However, future studies will need to probe the trend of PWB during the future different phases and after the end of the COVID-19 pandemic, and to replicate the survey in other settings or countries, in particular investigating the interaction between patterns of cultural access and other factors known to affect the PWB.

## Supplementary Information

Supplementary Information.

## Data Availability

The datasets used and/or analysed during the current study available from the corresponding author on reasonable request.
