# Supplementary material for: Impact of COVID-19 emergency on the psychological well-being of susceptible individuals
Source: Sci Rep. 2022 Jul 1;12:11152. doi: 10.1038/s41598-022-15357-6 (PMC9247931; doi:10.1038/s41598-022-15357-6)
Supplement: Supplementary file 1 — Supplementary Information. [file 41598_2022_15357_MOESM1_ESM.docx]

**IMPACT OF COVID-19 EMERGENCY ON THE PSYCHOLOGICAL WELL-BEING OF SUSCEPTIBLE INDIVIDUALS**

Angela Stufano, MD,^1a^ Guglielmo Lucchese, MD PhD,^2a^ Benjamin Stahl, PsyD PhD,^2,3,4,5^ Ignazio Grattagliano, PsyD,^6^ Liliana Dassisti, PsyD,^6^ Piero Lovreglio, MD PhD,*^1^ Agnes Flöel, MD,^2^ Ivo Iavicoli, MD PhD, ^7^

***Supplementary informations***

**Questionnaire**

**"ASSESSMENT OF SUSCEPTIBLE WORKER STATUS IN COVID-19 EMERGENCY"**

Family name: ………………………………..… First name: …….………………………

Place of birth: ……………………………..…… Date of birth: ………………………….

**WORK HISTORY**

Job: …………………………………………………………………………………

Working seniority: ……………………………………………………………

| Planned activities for the job |  |  |  |  |
| --- | --- | --- | --- | --- |
| PROXIMITY(< 2m) | | | | |
| Alone |  |  |  |  |
| with others but not in proximity |  |  |  |  |
| with others in shared spaces but with adequate distancing |  |  |  |  |
| with others for a non-prevalent part of the time |  |  |  |  |
| in close proximity most of the time |  |  |  |  |
| AGGREGATIONS | | | | |
| Limited or no presence of third parties |  |  |  |  |
| Intrinsic presence of third parties but organizationally controllable |  |  |  |  |
| controllable with procedures |  |  |  |  |
| limited control despite procedures |  |  |  |  |

PHYSIOLOGICAL HISTORY

Weight ………………. Height ………………… BMI ……………..

Drug therapy: □No □Yes………………………………………………………………

ALCOHOL CONSUMPTION

□None □Occasional □Daily

N Units |__|__| /day: |__|__| n°Unit |__|__| /week: |__|__|

SMOKING HABIT

□ Non smoker □ Smoker □ Previous smoker

Starting age of smoking habit |__|__| N. of packs/year |__|__|

**RESPIRATORY RISK**

□ COPD

COPD Assessment Test (CAT)

For each item reported use score 0 (no condition) to 5 (condition always present)

| I never cough | 0 | 1 | 2 | 3 | 4 | 5 | Cough always |
| --- | --- | --- | --- | --- | --- | --- | --- |
| Absence of expectoration | 0 | 1 | 2 | 3 | 4 | 5 | Presence of expectoration |
| I do not feel any tightness in my chest | 0 | 1 | 2 | 3 | 4 | 5 | I have a strong feeling of constriction in my chest |
| When I walk uphill or climb a flight of stairs I do not feel short of breath | 0 | 1 | 2 | 3 | 4 | 5 | When I walk uphill or climb a flight of stairs I feel very short of breath |
| I feel comfortable leaving the house despite my lung disease | 0 | 1 | 2 | 3 | 4 | 5 | I don't feel comfortable leaving the house at all because of my lung disease |
| I sleep deeply | 0 | 1 | 2 | 3 | 4 | 5 | I cannot sleep deeply because of my lung disease |
| I have a lot of energy | 0 | 1 | 2 | 3 | 4 | 5 | I have no energy |

□ ASTHMA

ASTHMA CONTROL TEST (ACT)

| SYMPTOMS IN THE PAST 4 WEEKS | | | | | |
| --- | --- | --- | --- | --- | --- |
| Daytime symptoms more than twice a week | Always | Very often | Sometimes | Rarely | Never |
| Nocturnal awakenings | Always | Very often | Sometimes | Rarely | Never |
| Bronchodilators needed more than twice per week | Always | Very often | Sometimes | Rarely | Never |
| Limitation of activities due to asthma | Always | Very often | Sometimes | Rarely | Never |

□ LUNG INTERSTITIAL DISEASES

□ Silicosis □ Asbestosis □ Sarcoidosis □ Other interstitial diseases……………………………….

□ DLCO………… □ SpO_2_ ………………. □ FVC □>75% □36-55% □≤35%

□**CARDIOVASCULAR RISK**

□Arterial hypertension □Hypertensive heart disease □Heart Failure □ Ischemic Heart Disease □ Atrial fibrillation □Valvulopathy□ Aortic Aneurysm □Obliterative arteriopathy□ Carotid Stenosis

□ **THROMBOEMBOLISM RISK (last five years)**

□ Venous Thrombosis □ Pulmonary Thromboembolism □ Platelets………….

□ Neutrophil/lymphocyte ratio……...□ Mutations associated with hypercoagulability…………

□ **NEOPLASTIC RISK (last five years)**

□ Neoplasm diagnosed < 1 year □ Neoplasm diagnosed 1-5 years before

□ Localized □ Metastatic □ TNM Classification ……………………..

Under treatment: □ cytostatic □ immunotherapy □ radiant

□ **NEUROLOGICAL RISK**

□ Alzheimer's disease/other dementias □ Parkinson's disease/Parkinsonisms

□ Motoneuron disease □ Idiopathic developmental intellectual disability

□ Chronic cerebrovascular disease □ Multiple Sclerosis

□ Stroke (past 5 years)

□ **RENAL RISK**

Chronic kidney disease secondary to □ diabetes □hypertension □ glomerulonephritis

□unspecified cause □ other causes …………………………………..

Glomerular filtration rate (GFR): □30-60 ml/min □< 30 ml/min □ Dialysis treatment

□ **DIABETOLOGICAL RISK**

□ Diabetes mellitus type 1 □ Diabetes mellitus type 2

Hb1Ac in the last year □ ≤58 mmol/mol □ > 58 mmol/mol

□ **LIVER RISK**

Cirrhosis/Chronic liver disease secondary/correlated to: □ HBV □ HCV □ Alcohol abuse

□ Autoimmune □ Other causes…………… □ Bilirubin……. □ AST/ALT ratio……. □αfetoprotein…….

□ **RISK RELATED TO AUTOIMMUNE DISEASE**

□ Psoriasis □ Systemic lupus erythematosus □ Rheumatoid arthritis

□ Chronic inflammatory bowel disease

□ **RISK RELATED TO INFECTIOUS DISEASE**

□ Tuberculosis (excluding latent forms without primary complex)

□ HIV-AIDS

□**RISK FROM IMMUNODEPRESSION**

□ Congenital immunodeficiencies □ Acquired immunodeficiencies

□ Hemopoietic stem cell transplantation □ Solid organ transplantation

□ Chronic Splenic Pathology/Splenectomy

**Immunosuppressive treatments**: □ Azathioprine □ Cyclophosphamide

□ Calcineurin inhibitors □ Methotrexate □ Mycophenolate □ Steroids (dose > 1mg/Kg)
